# Comparing high and low energy outcomes on day one for SmartSight myopic-astigmatism treatments with the SCHWIND ATOS: a retrospective case series

**DOI:** 10.1186/s12886-023-03076-z

**Published:** 2023-07-18

**Authors:** Kishore Raj Pradhan, Samuel Arba Mosquera

**Affiliations:** 1 Matrika Eye Center, Dhunge Dhara Marg, Ward number 9, Kathmandu, 44600 Nepal; 2 SCHWIND eye-tech-solutions, Mainparkstr. 6-10, Kleinostheim, 63801 Germany

**Keywords:** Myopic astigmatism, Lenticule, Lenticule extraction, Small incision, Femtosecond laser, Early outcomes, Immediate outcomes, Immediate and short-term visual recovery, Corneal laser refractive surgery, Smoothness

## Abstract

**Background:**

Impact of low energy asymmetric spacings vs. high energy symmetric spacings on the immediate/early (postoperative day 1 (POD1)) outcomes of SmartSight lenticule extraction for myopic astigmatism with a new femtosecond laser system.

**Methods:**

The first 112 eyes of 56 patients consecutively treated using low energy asymmetric spacings (Group A; Study group) were compared at POD1 to the last 112 eyes of 56 patients consecutively treated using high energy symmetric spacings (Group S; Controls). Mean age of the patients was 28 ± 5 years with a mean spherical equivalent of -4.41 ± 1.76 diopters (D) and a mean magnitude of refractive astigmatism of 0.89 ± 0.82 D.

**Results:**

Laser Energy was -25 ± 1nJ lower for asymmetric treatments (*p* < .0001); Spot and Track distances were + 0.7 ± 0.1 µm larger and -0.8 ± 0.1 µm tighter for asymmetric treatments, respectively (*p* < .0001 for both). At POD1, astigmatism was -0.08 ± 0.02D lower for asymmetric treatments (*p* < .0003); uncorrected and corrected visual acuities (UDVA and CDVA, respectively) were -0.03 ± 0.01logMAR better for asymmetric treatments (*p* < .0007); differences between postop UDVA and preop CDVA along with change in CDVA were + 0.3 ± 0.1lines better for asymmetric treatments (*p* < .0003).

**Conclusions:**

Lenticule extraction treatment using SmartSight is safe and efficacious already at POD1. Findings suggest that low energy asymmetric spacings may further improve the immediate and short-term outcomes of SmartSight lenticule extraction in the treatment of myopic astigmatism compared to conventional settings (high energy symmetric spacings).

## Background

Corneal refractive surgery is a form of vision correction consisting in the removal of corneal tissue (acting as a lens) to morphologically adapt the corneal geometry to correct for optical and visual defects.

Smoother corneal surfaces after corneal refractive surgery have related advantages, including better short-term outcomes for the time in which the epithelium remodeling (smoothing/masking) takes place; secondary to that, the time for surface recovery may be shorter (since the epithelium may undergo less remodeling), both resulting in reduced post-operative discomfort for the patient. It has been suggested and hypothesized, that better final vision and reduced levels of induced Higher Order Aberrations (HOAs) may also result from a smoother corneal surface [1, 2].

SmartSight treatment (SCHWIND eye-tech solutions GmbH, Kleinostheim, Germany) is a lenticule creation and extraction implemented in the SCHWIND ATOS femtosecond system (SCHWIND eye-tech solutions GmbH, Kleinostheim, Germany) [3, 4].

The aim of this retrospective case series is to investigate immediate and short-term (at postoperative day 1, (POD1)) visual recovery in myopic eyes (with or without astigmatism) treated with SmartSight procedure. An additional aim of this study was to compare the impact of low energy asymmetric spacings vs. conventional high energy symmetric spacings on the immediate/early (POD1) outcomes of SmartSight lenticule extraction for myopic astigmatism with a new femtosecond laser system.

The purpose and meaning of this work may help improving the laser energy and spot spacings design to achieved better outcomes in laser driven lenticule extraction procedures.

## Methods

The general methodology applied in this work is similar to the the methods described in a previous publication [5]. The main difference being its application to a cohort of patients operated using corneal lenticule extraction procedures, instead of transepithelial PRK treatments.

### Patients

This retrospective, observational study was based on a series of patients treated with the SmartSight technique to correct myopia with and without astigmatism, at the Matrika Eye Center, Kathmandu, Nepal. Before the procedure, patients were adequately informed about the risks and benefits of the surgery. All patients signed informed consent form (ICF) in accordance with the Declaration of Helsinki, for both the treatment and use of their de-identified clinical data for publication. The study has been evaluated according to the National Ethical Guidelines for Health Research in Nepal (revised in 2022) by the Matrika Eye Center and deemed not to require ethics approval due to the retrospective nature of the review chart. The purpose of this clinical research does not represent a clinical investigation. The medical device was used within its intended purpose without any additional invasive or patient burdensome procedures used.

Inclusion criteria included: Subjects 18 years of age or older, able to comprehend and sign an ICF, stable refraction, discontinuation of the contact lenses prior to the preoperative evaluation. Patient charts had CDVA of 20/32 or better, stable refraction for more than 1 year prior to the study. Patients were required to have normal keratometry and topography (SIRIUS, CSO, Italy), including a calculated central residual stromal thickness of 275 µm or more as calculated/estimated by the device at the time of the plan. A total of 224 eyes of 112 consecutive patients were retrieved in the review chart.

The average age of the patients was 28 years ± 5 (range 18 to 44 years). The mean preoperative spherical equivalent was -4.41D ± 1.76 (-12.75 to -1.25D), with mean preoperative astigmatism 0.89D ± 0.82 (0 to 5.25D).

### Preoperative assessment

A full ophthalmologic examination was performed on all the patients prior to surgery including manifest refraction, cycloplegic refraction and corneal topography (SCHWIND Sirius). Corrected distance visual acuity (CDVA) and uncorrected distance visual acuity (UDVA) were assessed. The corrected visual acuity was assessed with trial frames and not contact lenses. All the tests were performed monocularly. Preoperatively, information on general and ocular medical history, contact lens wear, and medication use was obtained from each patient.

### Surgical procedure

All the treatments were prepared at the SCHWIND ATOS in Lenticule mode (SCHWIND eye-tech-solutions GmbH, Kleinostheim, Germany). The devices used in this study meet the standards of European conformity (Conformité Européene or CE marking) but are not approved by the U.S. Food and Drug Administration (FDA).

The sphere and cylinder values entered into the laser were based on the manifest refraction with nomogram adjustments based on the previous experience of the surgeon (adding up to -0.5D to the manifest sphere (depending on the age of the patients, the level of myopia, and the size of the optical zone, among others), but the analyses were performed as deviation from the planned correction, instead of from clinical target). Caps were 150 to 160 µm thick, the optical zone ranged from 5.5 to 7.0 mm, the incision was positioned pseudo-superior at 150° (irrespective of the laterality, OD-OS) with entry angle of 120° and an incision length between 2.5 and 3.0 mm. The optical zone selected depended on the scotopic pupil size and attempted correction. The SmartSight profile includes a refractive progressive transition zone (similar to the one used in the SCHWIND AMARIS) of up to 0.8 mm (depending on the corneal curvature gradient otherwise induced by the correction) [6] tapering the lenticule towards the edge of the transition zone, without the need of a minimum-lenticule-thickness pedestal [7]. For each treatment, the system calculated the size of the optimal transition zone, depending on the treatment refraction and optical zone. All eyes underwent the refractive treatment using a 5.8 to 7.7 mm lenticular diameter.

Before the surgery, proparacaine hydrochloride 0.5% drops (Alcaine, Alcon, USA) were instilled in the upper and lower fornices 3 times within a 5-min interval. A sterile drape covering eye lashes was used to isolate the surgical field. A lid speculum was inserted to allow maximum exposure of the globe.

After placing the patients on the surgical bed and administering topical anaesthesia, patients were instructed to fixate on the fixation light to help centration [8]. SCHWIND ATOS femtosecond laser (SCHWIND eye-tech-solutions, Kleinostheim, Germany) was used for the SmartSight procedure. After the patient was positioned on the bed, the cone (a disposable patient interface with a curved contact glass) was inserted in the system. The patient’s eye was positioned under the cone and patient was instructed to fixate the light target.

Accurate alignment of the eye with the laser was achieved with an infrared eye tracker with simultaneous limbus, pupil, and torsion tracking integrated into the laser system and centred on the corneal vertex. The correction profile was cantered on the corneal vertex determined by the topography (taking the pupil offset value [9]), which closely approximates the visual axis [10, 11].

In order to accomplish centration and docking of the eye to the system, an eye-tracker guided centration is used [5]. A camera provides the operator with a coaxial view through the cone of the patient interface. The operator is instructed to move the patient table and bring the pupil (detected by the video-based Eye-Tracker) coincident with the target pupil position (or as close as possible). Then suction was applied at a level of 250 mmHg (vacuum level, and not intraocular pressure), and it was confirmed that the pupil remained close to its target position; otherwise, a new docking was attempted. Further to that, the last valid laser videoframe of the eye-tracker has been used for cyclotorsion control, and the torsional misalignment from the diagnostic image has been determined and accounted for [12]. The treatment was applied, and the laser ablation initiated after suction.

A single surgeon (KRP) performed the treatments, using an identical surgical protocol. In all cases, automatic cyclotorsion control was verified before the surgery (but no dynamic cyclotorsion control is included after suction).

For the SmartSight procedure, SCHWIND ATOS works in the low-density plasma region [13], above the threshold for laser induced optical breakdown [14], but below the photodisruption regime [15]. In this series pulse energies between 80 and 120nJ have been used.

Once the lenticule creation was completed, suction was released automatically. A thin blunt spatula was inserted through the incision to first identify two sides of the lenticule and then separate the lenticule (first the anterior and then the posterior surface) from the stroma and extracted through the incision. After extraction, cornea was gently massaged (ironed) in straight movements from the 6 o’clock position towards the incision in order to spread the cap evenly and potentially decrease Bowman’s wrinkles [16]. In the end, the remaining tissue was checked for any residual material or tears.

### Postoperative evaluation

For this cohort, only the preoperative and the POD1 postoperative examination visit was retrieved. POD1 postoperative examinations included UDVA, CDVA, manifest refraction, and slit lamp examination. The postoperative therapy was the same for all patients.

### Comparison of low energy asymmetric spacings vs. high energy symmetric spacings

Further analysis was performed to compare eyes treated with low energy asymmetric spacings vs. eyes treated with high energy symmetric spacings, in terms of their achieved visual acuity at POD1 after performing SmartSight procedure.

The first 112 eyes of 56 patients consecutively treated using low energy asymmetric spacings (Group A; Study group) were compared at POD1 to the last 112 eyes of 56 patients consecutively treated using conventional high energy symmetric spacings (Group S; Controls).

### Statistical analysis

Visual acuity was evaluated in logMAR. The analysis comprised evaluating the change in visual acuity for all the eyes, preoperatively vs. at POD1. The normality of the samples has been estimated using the back-of-the-envelope test for sample size of 112 eyes (leading to a 2.6SD estimate). Paired Student’s t-tests were used to evaluate the difference between preoperative and postoperative visual acuity. Unpaired tests were used to evaluate the difference between groups (Student’s t-tests for mean values and Fisher’s exact test for percentages). A *p* value less than 0.05 was considered statistically significant.

## Results

**Fig. 1:**
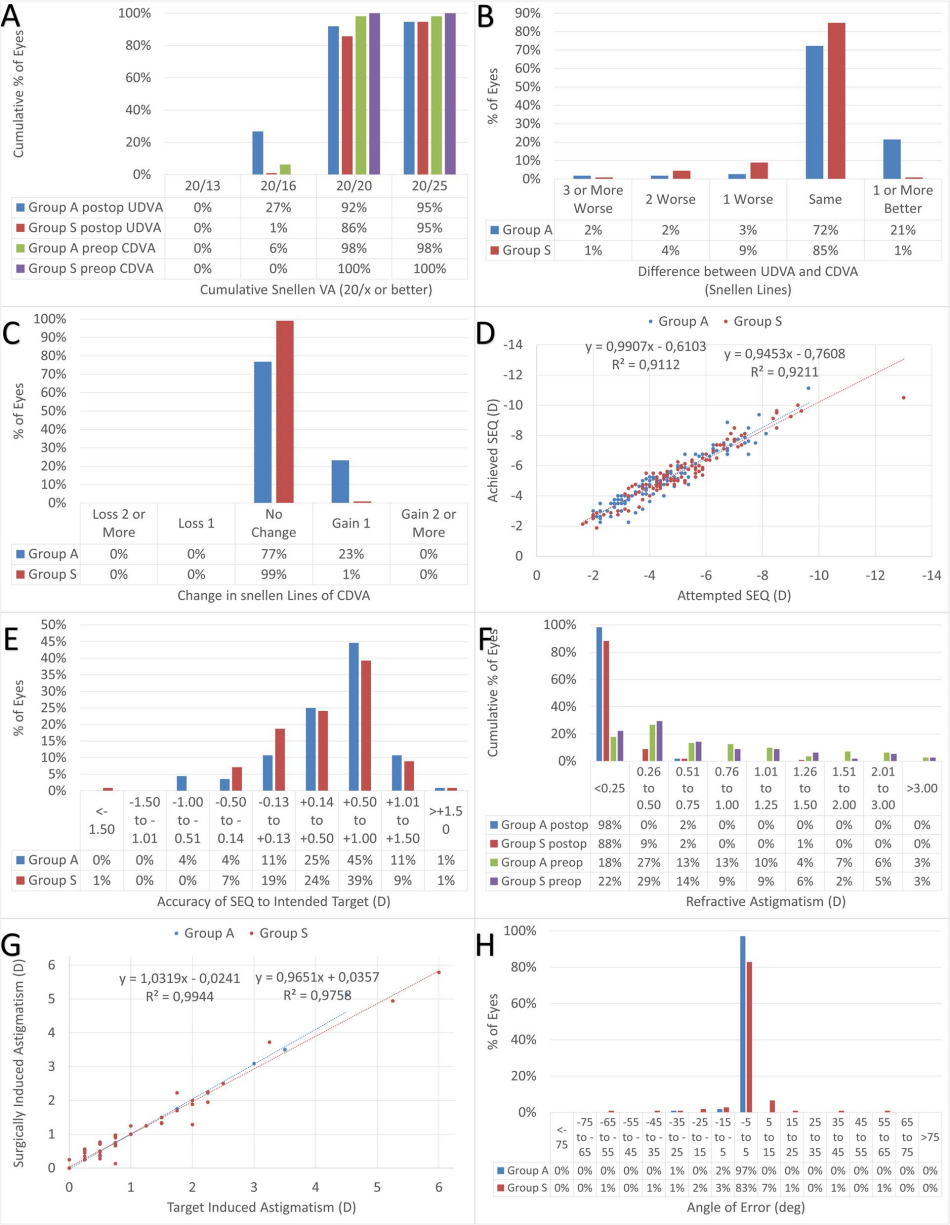
Standard graphs for reporting astigmatism outcomes of refractive surgery at POD1 comparing the first 112 eyes of 56 patients consecutively treated using low energy asymmetric spacings (Group A; Study group) vs. the last 112 eyes of 56 patients consecutively treated using high energy symmetric spacings (Group S; Controls). **A** Cumulative Snellen Visual Acuity. **B** Difference between achieved UDVA at POD1 and preoperative CDVA baseline. **C** Change in Snellen lines of CDVA. **D** Scattergram of the SEq. **E** Predictability of the SEq respect to the target. **F** Predictability of the refractive astigmatism respect to the target. **G** Scattergram of the astigmatic correction. **H** Predictability of the refractive astigmatic axis respect to the target

Table 1 provides a summary of the intrinsic and extrinsic parameters retrospectively retrieved for both groups. All 224 eyes completed Day 1 follow up. POD1 standard graphs for reporting astigmatism outcomes are presented in Fig. 1.

**Table 1:** Summary of the intrinsic and extrinsic parameters retrospectively retrieved for both groups

	**Group A (low energy asymmetric spacings; Study group)**		**Group S (high energy symmetric spacings; Controls)**		***p*-value**
	mean ± StdDev	Range	mean ± StdDev	Range	
**n**	112	---	112	---	---
**Date of Birth**	6/Jun/1993 ± 5 years	18/Dec/1978 to 3/Jun/2004	13/Oct/1994 ± 4 years	08/Jun/1984 to 12/Jun/2004	.02
**Gender (M:F)**	33:23	---	30:26	---	.2
**Eye (OD:OS)**	28:28	---	28:28	---	.5
**Treatment Date**	17/Jul/2022 ± 11 days	03/Jul/2022 to 05/Aug/2022	8/Dec/2021 ± 222 days	8/Nov/2020 to 30/Jun/2022	< .001
**Age (y)**	29 ± 5	18 to 44	27 ± 5	18 to 38	.002
**Preoperative SEq (D)**	-4.17 ± 1.59	-1.63 to -9.88	-4.65 ± 1.92	-1.25 to -12.75	.1
**Preoperative Ast (D)**	0.93 ± 0.78	0 to 4.00	0.85 ± 0.87	0 to 5.25	.2
**Preop CDVA (logMAR)**	0.0 ± 0.1	-0.1 to + 0.2	0.0 ± 0.0	0.0 to 0.0	.4
**Target Sph (D)**	+ 0.96 ± 0.33	-1.00 to + 1.37	+ 0.95 ± 0.37	-2.00 to + 1.50	.4
**Planned SEq (D)**	-4.53 ± 1.64	-2.00 to -9.63	-5.00 ± 1.88	-1.63 to -13.00	.1
**Planned Cyl (D)**	0.98 ± 0.78	0 to 4.50	0.91 ± 0.93	0 to 6.00	.3
**Flat Keratometry (D)**	43.0 ± 1.3	40.0 to 46.5	42.8 ± 1.4	39.5 to 45.0	.2
**Steep Keratometry (D)**	44.2 ± 1.5	40.9 to 47.8	43.9 ± 1.4	40.2 to 46.7	.1
**Central Pachymetry (µm)**	535 ± 25	488 to 580	536 ± 30	427 to 633	.3
**Central Epithelium (µm)**	51 ± 3	44 to 56	51 ± 3	42 to 58	.3
**Planned OZ (mm)**	6.0 ± 0.2	5.5 to 6.5	6.0 ± 0.2	5.5 to 7.0	.1
**Lenticule Diameter (mm)**	6.8 ± 0.2	5.8 to 7.3	7.0 ± 0.4	6.3 to 7.7	.04
**Cap diameter (mm)**	7.8 ± 0.2	7.4 to 8.3	7.9 ± 0.3	7.3 to 8.7	.02
**Cap Thickness (µm)**	160 ± 0	160 to 160	156 ± 5	150 to 160	< .001
**Laser energy (nJ)**	92 ± 13	80 to 115	118 ± 3	110 to 120	< .001
**Spot Distance (µm)**	4.4 ± 0.3	4.1 to 4.7	3.7 ± 0.3	3.2 to 3.9	< .001
**Track Distance (µm)**	2.9 ± 0.5	2.5 to 3.8	3.7 ± 0.3	3.2 to 3.9	< .001
**Treatment Dose (mJ/cm**^**2**^**)**	733 ± 12	716 to 772	790 ± 12	789 to 887	< .001
**Laser Avg. Power (mW)**	66 ± 13	57 to 88	100 ± 6	97 to 113	< .001
**Incision Location (deg)**	150 ± 0	150 to 150	150 ± 0	150 to 150	–-
**Incision Length (mm)**	2.5 ± 0.1	2.5 to 3.0	3.0 ± 0.0	3.0 to 3.0	< .001
**Incision Angle (deg)**	120 ± 0	120 to 120	120 ± 0	120 to 120	–-

The following parameters were not strictly following a normal distribution as determined by the back-of-the-envelope test: magnitude of the astigmatism, magnitude of the corneal toricity, target sphere, diameter of the OZ, postoperative CDVA, change from preoperative CDVA to postoperative UDVA, change in central corneal thickness, and angle of error.

### Overall POD1 outcomes

Overall, 89% of the eyes reached UDVA of 20/20 or better at POD1, with 14% in 20/16 or better (Fig. 1A). Overall, 96% of the eyes achieved a UDVA at POD1 within a line from preoperative CDVA baseline (Fig. 1B). Overall, 12% of the eyes gained lines of CDVA at POD1; whereas no eyes lost lines of CDVA (Fig. 1C). Scattergram of the SEq correction showed a ~ 0.5D overcorrection (Fig. 1D), with the overall predictability of 58% of the eyes within 0.75D of the attempted SEq at POD1 (Fig. 1E). Scattergram of the astigmatic correction showed no overcorrection or undercorrection (Fig. 1G), with the overall predictability of 97% of the eyes with less than 0.5D astigmatism at POD1 (Fig. 1F) and 90% of the eyes within 5 deg of the attempted astigmatism axis (Fig. 1H).

### Intergroup differences between low energy asymmetric spacings vs. high energy symmetric spacings

Date of birth was 1.3 ± 0.7y older for asymmetric treatments (*p* < 0.03); Age was 2.0 ± 0.7y older for asymmetric treatments (*p* < 0.002). Treatment date was 221 ± 21 days later for asymmetric treatments (*p* < 0.0001).

Laser pulse energy was -25 ± 1nJ lower for asymmetric treatments (*p* < 0.0001); spot distance was + 0.7 ± 0.1 µm larger for asymmetric treatments (*p* < 0.0001); track distance was -0.8 ± 0.1 µm shorter for asymmetric treatments (*p* < 0.0001).

Incision length was -0.5 ± 0.1 mm shorter for asymmetric treatments (*p* < 0.0001).

Postop cylinder was -0.08 ± 0.02D lower for asymmetric treatments (*p* < 0.0003); achieved change in corneal toricity was 0.31 ± 0.11D higher for asymmetric treatments (*p* < 0.005).

Postop UDVA was -0.03 ± 0.01logMAR better for asymmetric treatments (*p* < 0.0007); postop CDVA was -0.02 ± 0.01logMAR better for asymmetric treatments (*p* < 0.0002). The difference between postop UDVA and preop CDVA was + 0.3 ± 0.1lines better for asymmetric treatments (*p* < 0.0003); the change in CDVA was + 0.2 ± 0.1lines better for asymmetric treatments (*p* < 0.0001).

## Discussion

The aim of this study was to investigate immediate and short-term visual recovery in myopic eyes (with or without astigmatism) treated with SmartSight procedure. In this retrospective case series, the post-operative outcomes were evaluated immediately after the surgery (at day 1, POD1) after myopic SmartSight treatment. An additional aim of this study was to compare the impact of low energy asymmetric spacings vs. high energy symmetric spacings on the immediate/early (POD1) outcomes of SmartSight lenticule extraction for myopic astigmatism with a new femtosecond laser system.

The general methodology applied in this work is similar to the the methods described in a previous publication [5]. The main difference being its application to a cohort of patients operated using corneal lenticule extraction procedures, instead of transepithelial PRK treatments.

For detecting short term differences between two different settings, it is expected that the maximum difference can be observed directly immediately after the extraction before the onset of epithelial remodeling [5]. This difference will likely decrease with time, due to the masking effect induced by the epithelial repopulation (and remodeling). To capture these early effects in outcomes, visual acuity is reported for POD1 in this work, instead of longer follow up results (commonly seen in LVC reports). A 3-days follow-up report of UDVA may have avoided confounding effects due to acute oedematic reactions; yet (at the light of the observed and reported UDVA) corneal oedema was minimum (or at least comparable) in both groups. Further, 3-days is not a scheduled follow-up visit in the clinic. On the other hand, a 1-week follow-up was considered “too long” for the aim of this work, and potentially some of the underlying differences may have already been masked by the epithelial remodeling at 1-week. As an “informal” sanity-check [5], the subsequent follow-ups of patients from either group were unremarkable, and overall results tend to remain stable (or even improve for both groups) over the first 12-months of follow-up [3, 4]. The differences between groups tend to reduce with time (but the advantage of the asymmetric settings appears to be present in a “diluted” manner).

The positive impact of the change in laser settings was seen in terms of the quick recovery in visual acuity after the SmartSight treatments in our cohort; with the achieved POD1 average UDVA being 2 optotypes better for the low energy asymmetric spacings, with a higher proportion of eyes reaching the 20/20 and 20/16 levels of UDVA at POD1. In our cohort, at POD1, the eyes treated with the low energy asymmetric spacings performed slightly better in terms of astigmatic correction, as well.

Similar studies have been published in the past to evaluate the early short-term visual acuity in SMILE. Recchioni et al. [17] evaluated the early clinical outcomes after the first small incision lenticule extraction (SMILE) cases undertaken by three surgeons at a single site in the UK. The results showed that 88% achieved 20/20 or better uncorrected distance visual acuity at three months. In comparison, in our cohort where SmartSight was performed, 89% eyes achieved monocular UDVA 20/20 or better already at POD1.

The early time course of the visual recovery after small incision lenticule extraction (SMILE) and laser in situ keratomileusis (LASIK) has been reported by Chicha et al. [18]. The LASIK group showed better contrast sensitivity at 1 day (*P* = 0.014) and 7 days (*P* = 0.001) but not at 1 month. Similalry, at 1 day postoperatively LASIK produced a lower objective scatter index assessed by double-pass aberrometry (*P* = 0.036), yet this was not statistically different thereafter, whereas at 7 days, the SMILE group self-reported a worse quality of vision than the LASIK group (*P* = 0.010).

Kamiya et al. [19] investigated the early clinical outcomes of small incision lenticule extraction (SMILE) to correct both myopia and myopic astigmatism at major clinical centers in Japan. Liu et al. [20] compared visual acuity between SMILE and FS-LASIK evaluated at 0, 2, 4 and 24 h postoperatively. At 2 h and 4 h after surgery, visual acuity scores in the SMILE group were significantly poorer than those in the FS-LASIK group (*p* < 0.05). Tay and Bajpai [21] assessed visual recovery after small incision lenticule extraction (SMILE) in relation to pre-operative spherical equivalent. 41% eyes had uncorrected distance visual acuities of 20/20 at 1 day, 72% at 2 weeks, 91% at 1 month and 93% at 3 months. Significantly more eyes with low myopia (up to -5D) achieved acuities of 20/20 at 1 day and 2 weeks (*p* = 0.041 and *p* < 0.001). Post-operative acuities were not associated with refractive targets, laser cut energy settings or other variables. In comparison, in our overall cohort, 89% eyes achieved UDVA 20/20 or better at POD1.

Donate and Thaëron [22] were the first to propose low energy levels to enhance early visual recovery after lenticule extraction. In their study, spot and track distances remained symmetric (4.5 µm for both) and pulse energy was reduced from 180nJ down to 100nJ for the study group (with better early recovery). This simple measure actually reduced not only pulse energy but simultaneously treatment dose (from 0.9 J/cm^2^ down to 0.5 J/cm^2^) and laser average power. In our cohort, as well, not only pulse energy but simultaneously treatment dose (from 0.8 J/cm^2^ down to 0.7 J/cm^2^) and laser average power (from 100mW down to 66mW) were also reduced, yet to a much lower extent. As intuitively expected, this leads in fact to longer laser times (+ 25% to + 30%, representing less than 5 s extra time).

Hamilton et al. [23] compared low-energy (LE) vs. high-energy (HE) small-incision lenticule extraction (SMILE) and femtosecond laser-assisted laser in situ keratomileusis (FS-LASIK) procedures in terms of uncorrected distance visual acuities (UDVAs) in the early postoperative period. The LE group achieved a highly statistically significant better UDVA at POD1 compared to the HE group in SMILE patients (-0.003 vs 0.141, *P* < 0.0001), whereas no significant difference in mean UDVA at POD1 was noted between the LE group and FS-LASIK group (-0.003 vs -0.011, *P* = 0.498).

Donate and Thaëron [24], and Ji et al. [25, 26] further confirmed the benefit of reducing energy levels. Unlike our cohort, all these studies used pulse energies above 100nJ, and symmetric settings (Spot distance = Track distance). Asymmetric settings and pulse energies below 100nJ have been theoretically introduced recently [27].

A comparison of biomechanical effects of small-incision lenticule extraction and laser in situ keratomileusis was reported by means of a finite-element analysis [28]. Authors found that small-incision lenticule extraction may present less biomechanical risk to the residual bed of susceptible corneas than comparable corrections involving LASIK flaps and postulated that deeper corrections in the stroma might be possible in small-incision lenticule extraction without added risk for ectasia.

In the current cohort (this work), asymmetric treatments performed better for postop UDVA (-0.03 ± 0.01logMAR); postop CDVA (-0.02 ± 0.01logMAR); difference between postop UDVA and preop CDVA (+ 0.3 ± 0.1lines); and Change in CDVA (+ 0.2 ± 0.1lines).

It can be argued whether the nomogram adjustment (the same for both groups) was made to meet the patient satisfaction at POD1 but may hinder the long-time results requiring to be presented together with long term results. We shall acknowledge that the POD1 data does not necessarily lead to clinically meaningful differences later on. Thus, emphasizing the interest in longer term data. Yet, the emphasis of this work lies in the comparison between groups, and this comparison is irrespective of the stability or evolution of the outcomes. As a sanity check, the observed differences at POD1 between these 2 groups tend to reduce for longer follow-ups, while maintaining some advantages for the asymmetric settings group (unpublished data consistent over 1250 treatments). A similar behaviour (with advantages only observed at the shorter term follow-up) has also been reported for previous comparisons of low energy settings of another lenticule extraction technique [22, 24–26]. This can be explained by longer-term improvements in vision for the higher energy settings (showing lower vision in the short term, presumably due to a rougher interface, probably inducing a longer epithelium remodeling) [29] This has been previously reported also for surface ablation techniques [30] and apparently some degree of superior vision can be measured longer time [1].

From our presented outcomes, and at the light of the previous literature body, it can be inferred by exclusion that the observed improvement can neither be explained by the age differences (too small to render differences in such a moderate cohort, and opposed to what has been published before [31]), nor by the differences in lenticule diameter (too small to render differences in such a moderate cohort, and opposed to what has been published before [32]), nor by the differences in cap diameter or thickness (too small to render differences as it has been published before [33]), nor by the differences in incision size [34], nor by the differences in laser average power (no reports have been found comparing or reporting differences in the 200- vs. 500-kHz versions of one of the alternative lenticule extraction systems), nor by the differences in treatment dose (too small to render differences in such a moderate cohort). Thus, only the differences laser pulse energy “alone” (as previously reported), the differences in spot or track distances (i.e., the asymmetric spacing), or a combination of both can explain the improvement in short term visual acuities. Even if the pulse energy “alone” would be the key to better outcomes, the use of asymmetric spacings allows to further reduce the pulse energy (while maintaining overall dose and tissue separation)﻿ [27].

There are several limitations and potentially confounding factors in this study. Date of birth and accordingly age were 2 ± 1y older for asymmetric treatments. This difference is probably below clinical relevance and cannot be accounted for the observed differences. Both eyes of the patients were included, instead of randomly including only one eye from each patient. To account for this, we used the number of patients (instead of the number of eyes) to determine statistical significance.

Incision length was -0.5 ± 0.1 mm shorter for asymmetric treatments. This significant difference may account for the observed differences in astigmatic correction. Postop cylinder was -0.08 ± 0.02D lower for asymmetric treatments; achieved change in corneal toricity was -0.31 ± 0.11D higher for asymmetric treatments. Although, previous reports did not find differences respect to the incision size [34].

The repetition rate of the treatment laser pulses may also play a role if the lifetime of the cavitation bubble is longer than the interpulse time. Such effects can be inferred from previous works (at least for pulse energies well above the breakdown threshold) [35–38].

In fact, the repetition rate of the treatment laser pulses is crucial for intrastromal dissection in refractive surgery. The bubble remains within the cornea for a few microseconds. Thus, below ~ 50 kHz repetition rate would probably not play a relevant role, but beyond ~ 500 kHz repetition rate interactions with previous bubbles have to be considered. Asymmetric settings, as in our case (for which spot distance > track distance), come to play, since it results in lower linear and temporal overlap of the fast scanned pulses, combined with higher track overlap in a longer time domain. Thus, the local effective repetition rate is reduced compared to symmetric settings and the pulses have longer time and “room” to fully develop into cavitation bubbles.

In addition, closely neighboring pulses can strongly influence each other: A subsequent pulse may have to interact with material optically changed by the previous pulse, thus causing varying conditions for the laser-tissue interaction [39]. This aspect makes also asymmetric settings (using spot distance > track distance) better suited for an increased cutting efficiency and depth accuracy.

In this cohort laser pulse energy was -25 ± nJ lower for asymmetric treatments and the spacings asymmetric; thus, we cannot elucidate whether differences are related to energy, asymmetric spacings, or a combination of both. Yet, despite the large reduction in pulse energy, the asymmetric spacings were selected such that the treatment dose did not change much (although it reduced slightly in a statistically significant manner).

Other systems also work using sub 100nJ pulse energies [40]. Izquierdo et al. operated five eyes with a complete dissection and removal of the lenticule without intraoperative complications. In addition, at postoperative day 1, all patients had a clear cornea.

For symmetric settings (spot distance = track distance), the optimum pulse energy occurs for 3 × the threshold for laser induced optical breakdown (LIOBTh), whereas for asymmetric settings (as in this case, spot distance > track distance), the optimum pulse energy reduces to 1.5xLIOBTh. This means that for asymmetric settings, the optimum energy is just half of that required for symmetric settings in the otherwise same system.

The cutting resolution is also made more stable for the optimum criteria as the bubble size saturates quickly after optimum. Furthermore, the energy optimized for minimum dose shall reduce the thermal effects induced in the tissue, as well as the incidence of clinical complications as e.g., opaque bubble layers [41].

Unfortunately, and due to the retrospective nature of this work, we cannot provide a formal analysis or between groups comparison of the incidence and severity of OBLs. Anecdotally, it seems that the asymmetric settings also helped in this regard. But a study design may be specifically developed to address this point.

The energy regime above optimum (i.e., relative energy fluctuations are “attenuated” in smaller relative deviations in the size of the bubble) sets the energy sufficiently above threshold as to reduce the incidence of clinical complications as e.g., black spots [42].

Upon availability and due to the initial positive experience with low energy asymmetric settings, it became the treatment of choice at our practice for SmartSight procedures. Currently, we are using systematically low energy asymmetric settings of 85nJ in 4.7 × 2.5 µm distance (0.7 J/cm2 and 57mW) as primary settings; followed by 80nJ in 4.4 × 2.5 µm distance (0.7 J/cm2 and 57mW).

In this work, for some eyes emmetropia was not the intended target of the surgery. This may certainly affect (reduce) UDVA, which is the main evaluation criterion in this study and it could be argued that only eyes treated for emmetropia be analyzed. Actually, despite this expected reduction in UDVA for some eyes, the reported results showing a good short term visual recovery reinforce the benefits of the treatment. Further, the influence of the eyes having a non-emmetropic target is minor due to the overall number of treatments involved, and that slight hyperopic target refractions would account for the residual accommodation in this young population.

SmartSight treatment is a lenticule extraction procedure, implemented using the SCHWIND ATOS. An interesting question to pursue is whether SmartSight provides rapid visual recovery over other lenticule extraction techniques. We acknowledge that this comparison would add value to the results presented in this study (only comparing low energy asymmetric settings to high energy symmetric settings).

In order to analyze the early outcomes with SmartSight, we evaluated the visual recovery at POD1, because the maximum effect of the smoothness will be observable immediately after ablation, before epithelialization has begun. The best way to analyze if the low energy asymmetric spacings improve the immediate postoperative visual results after SmartSight should be a comparative eye-paired study, in which one eye should be treated with high energy symmetric spacings and the other eye of the same patient treated with low energy asymmetric spacings. Although, for a sample size of sufficient power, one could avoid the fellow-eye comparison, as we did.

To determine the sample size in a post-hoc attempt, the following values were taken: standard deviation (SD) of the postoperative UDVA (0.056 logMAR; 2.8 Optotypes). To detect a difference in UDVA barely reaching detectability (clinical relevance), the level of detection was set to 0.03 logMAR (1.5 Optotypes). The required sample size for α = 5% and 80% statistical power was 56 eyes per group. A total of 56 patients (112 eyes) per group (as in this comparison) satisfies that condition.

In addition, it would be advisable to compare not only the immediate visual recovery, but also the epithelial remodeling in both groups, in order to analyze if the SmartSight offers the advantage of a reduced epithelial healing or whether difference between low energy asymmetric settings and high energy symmetric settings are observed.

It would be interesting to analyze if the SmartSight (in asymmetric spacings) offers the advantage to induce lower HOAs, as compared to other lenticule extraction procedures (with symmetric settings) or using the same ATOS system and the same correction profile (i.e., SmartSight with symmetric spacings). We think focusing this work on short term visual acuity is a simple yet powerful topic, serving the demands of the patients (to regain UDVA as soon as possible regardless of the applied clinical technique).

Any statement on safety and efficacy at POD1 can be challenged if no formal analysis for longer term follow-ups is presented. Yet, in this cohort, POD1 outcomes were in both groups, at least as good as longer-term outcomes presented in the body of the literature. We cannot find any convincing reason (at the light of the literature on lenticule extraction reporting continuous improvements of the postoperative visual acuities over time) why this temporal behaviour could be different in our cohort. Our hypothesis (confirmed by informal observation) is that results are better from an earlier time point when using low energy asymmetric spacings and these outcomes remain stable over time.

## Conclusions

In our cohort of eyes, very early visual recovery (at POD1) after SmartSight was rapid, providing excellent UDVA immediately after the surgery. We postulate that it is the advanced myopic ablation pattern of the low energy asymmetric spacings used in the last group of SmartSight treatments that results in a smoother extraction and thus a more rapid visual recovery for patients. This work may help improving the laser energy and spot spacings design to achieved better outcomes in laser driven lenticule extraction procedures.

## Data Availability

The datasets analysed during the current study are not publicly available but are available (in a de-identified form) from the corresponding author on reasonable request.
